# Supplementary material for: Autoreactivity against Denatured Type III Collagen Is Significantly Decreased in Serum from Patients with Cancer Compared to Healthy Controls
Source: Int J Mol Sci. 2023 Apr 11;24(8):7067. doi: 10.3390/ijms24087067 (PMC10139183; doi:10.3390/ijms24087067)
Supplement: Supplementary file 1 [file ijms-24-07067-s001.zip › ijms-2290463-supplementary.pdf]

Supplementary Material

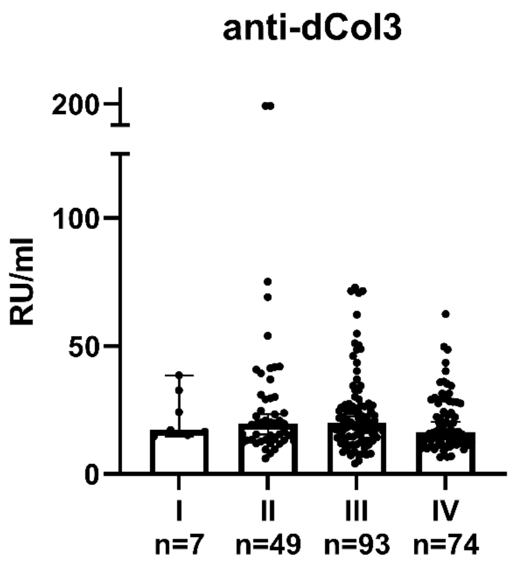

Figure S1. Anti-dCol3 in different cancer stages. Anti-dCol3 levels were measured in 223 patients with stage I (n=7), II (n=49), III (n=93) or stage IV cancer (n=74). Statistical differences were analyzed using the Kruskal-Wallis test adjusted for Dunn's multiple comparisons tests.

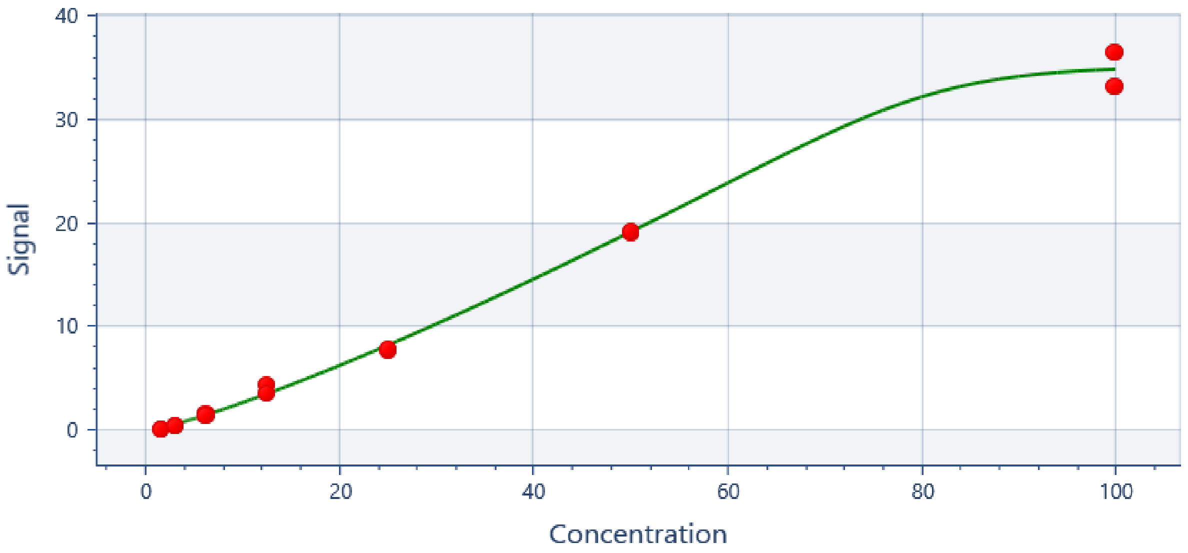

Figure S2. Standard curve. Anti-dCol3 levels were calculated based on a standard curve using a 5-parameter logistic curve fit y = a + ((b - (a)) / ((1 + (x/c)^d))^e).
